# Zenker's diverticulum misinterpreted as a thyroid mass: Case report

**DOI:** 10.1016/j.amsu.2020.11.004

**Published:** 2020-11-06

**Authors:** Fatemeh Salimi, Jerocin Vishani Loyala, Arbaaz Pervaiz, Stuart Winter

**Affiliations:** Oxford University Hospitals, NHS Foundation Trust, United Kingdom

**Keywords:** Zenker's Diverticulum, Neck Lump, Development of pharyngeal pouch, Surgical excision of the pharyngeal pouch

## Abstract

We discuss an unusual presentation of Zenker's Diverticulum (ZD). A 76-year-old man presented with a left sided neck mass which was misdiagnosed as a thyroid mass due to the anatomical location and size. The ultrasound and fine needle-aspiration cytology findings were inconclusive, and a CT scan was then considered which reported a large pharyngeal pouch. Our recommendation is to consider an early CT scan in patient's where there is a clinical suspicion or risk factors for the development of pharyngeal pouch specially when the fine-needle aspiration cytology findings are inconclusive. This would reduce the risk of a delayed diagnosis which can prevent potential perforation of the pharyngeal pouch and development of mediastinitis.

## Highlights

• Left sided neck mass misdiagnosed as thyroid mass.
• Zenker's Diverticulum is formed due to muscular weakness.
• Pharyngeal pouch symptoms mimic head and neck cancer presentation.
• Barium swallow is a gold standard investigation for pharyngeal pouch.
• Pharyngeal pouch can be treated with endoscopic or external surgical approaches.

## 1 Introduction

**Fig. 1:**
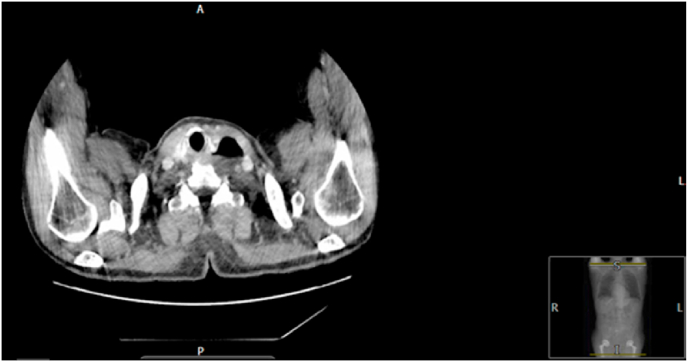
CT Contrast Neck, Left Pharyngeal pouch.

**Fig. 2:**
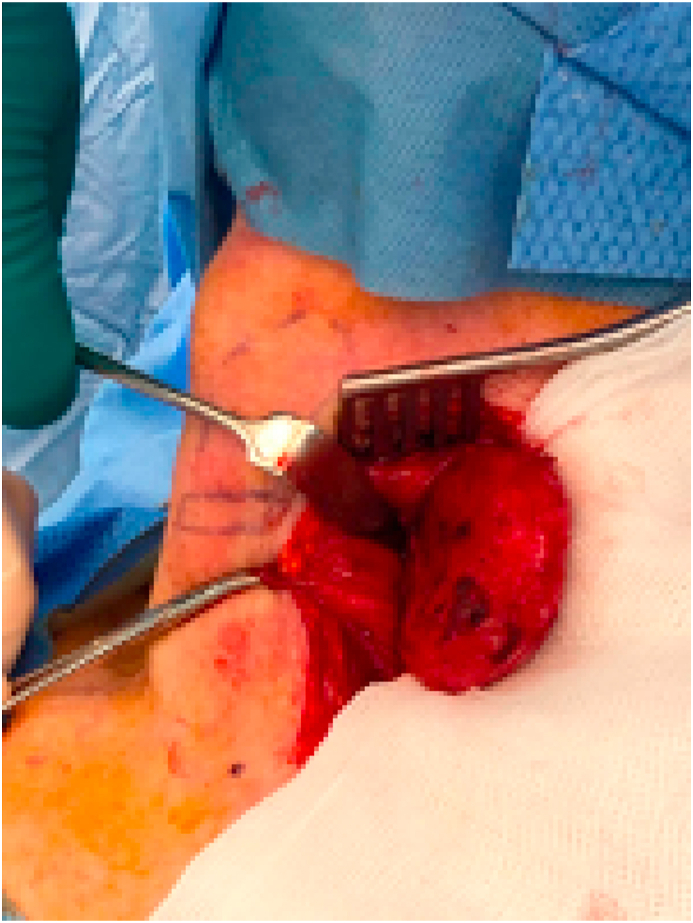
External approach to excise the pouch.

**Fig. 3:**
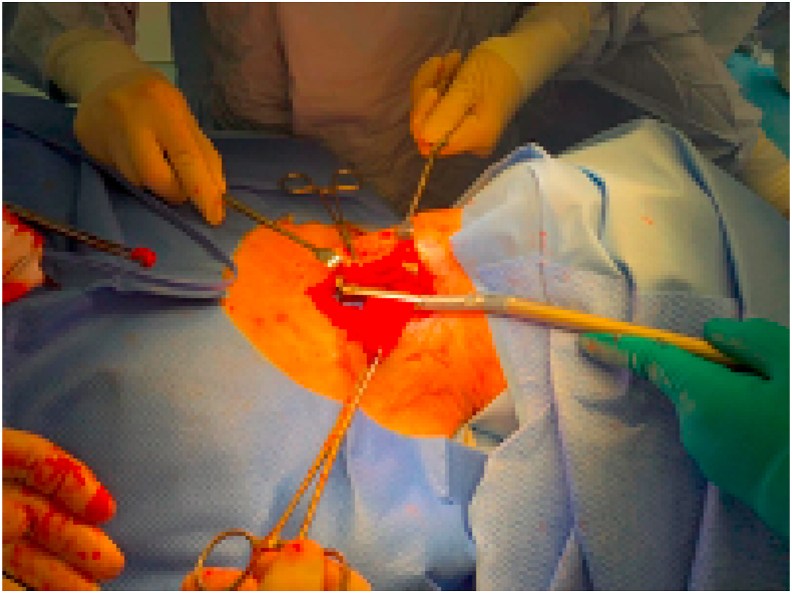
Stapling the pouch.

**Fig. 4:**
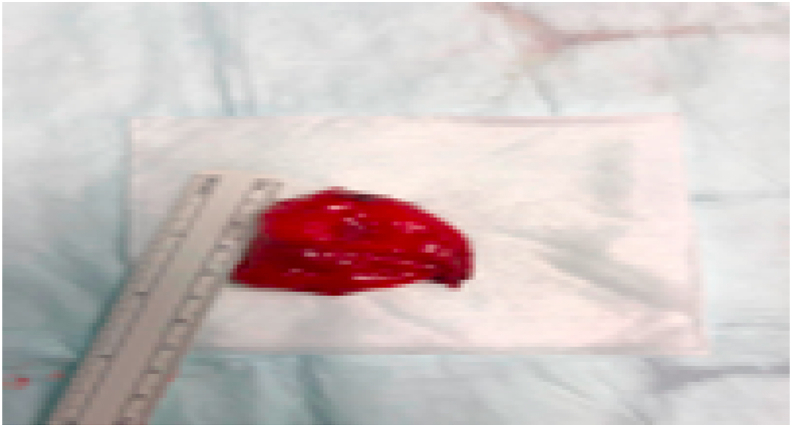
Excised Pharyngeal pouch, measuring 5cm in length.

Zenker's Diverticulum also known as a pharyngeal pouch is a diverticulum that occurs above the cricopharyngeal muscle, involving the pharyngeal mucosa and submucosa [1]. The pathophysiology underlying the development of ZD is still debatable and no definite cause of the mucosal outpouching has been defined. ZD's are more commonly seen in Northern Europe, USA and Canada and rarely in Asian countries such as Japan and Indonesia. In the UK the incidence of ZD is 2 per 100 000 people, however cases are considered to be under reported as patients can remain asymptomatic [2]. (see Fig. 1, Fig. 2, Fig. 3, Fig. 4)

ZD's are more commonly seen in males than females, and occur more frequently in the elderly population, especially in patient's over the age of 70. The diverticulum typically presents with various symptoms including chest pain, chronic cough due to aspiration, dysphagia, halitosis, regurgitation, and weight loss. As the pharyngeal pouch enlarges patients can become more symptomatic, malnourished, and often suffer from significant weight loss [1,3,4].

This case describes a distinctive presentation of a left sided neck mass which was mistaken as a thyroid mass in a patient who had prior endoscopic stapling of a pharyngeal pouch. The work has been reported in line with the SCARE criteria [13].

## 2 Case presentation

A 76-year-old man presented with a left sided neck mass, abdominal pain, progressive dysphagia over a few weeks and weight loss. He was referred urgently for investigation of a suspected thyroid malignancy under the two-week cancer referral pathway. He had a known past-medical history of dysphagia, total hip replacement, and nine years previously had endoscopic stapling for a pharyngeal pouch. On clinical examination, a left lateral level three neck mass was identified. The mass was described as approximately 4–5cm in diameter and palpable anterior to the sternocleidomastoid.

He was referred for an urgent ultrasound scan, which at the time reported a left sided thyroid nodule. This was described as a solid-cystic nodule, isoechoic with smooth margins and multiple echogenic foci with a comet tail artefact (in keeping with internal colloid) as well as multiple small foci of calcification. He then underwent a fine needle aspiration cytology (FNAC), the result of which was inconclusive. Based on the ultrasound findings he underwent a CT scan of his neck which showed a large (65 × 53 × 37mm) left sided pharyngeal pouch which was filled with more debris than gas.

Given the patient's progressive symptoms, surgical options for treating his pharyngeal pouch were discussed. He subsequently underwent external diverticulectomy. The final histology showed squamous epithelium lining with focal ulceration and patchy chronic inflammation in the subepithelial tissue. Following the procedure his dysphagia improved significantly enabling him to return to a normal diet.

This patient had an exceptionally large pharyngeal pouch measuring (65 × 53 × 37mm) radiologically which required external excision.

## 3 Discussion

A clear understanding of the pathogenesis underlying development of a pharyngeal pouch is missing. The most accepted theory is based on a physiological abnormality above the cricopharyngeal muscle at the level of the upper oesophageal sphincter [3,4]. This anatomical location is called Killian's triangle and pharyngeal pouch is also known as a Killian's dehiscence [3,5] which is an area of muscular weakness between the transverse cricopharyngeal fibres and oblique fibres of thyropharyngeal muscle. This triangle is predisposed to mucosal herniation.

This theory is further supported by malfunctioning of the cricopharyngeal muscle, the inability of the sphincter to relax and causing a raised hypopharyngeal (intrabolus) pressure during swallowing. The raised intrabolus pressure can be confirmed using specialized cricopharyngeal pH manometry [[3], [4], [5], [6]]. The intrabolus pressure is significantly higher in patients with a diverticulum in comparison to aged matched population without a pharyngeal pouch. The incidence of recurrence of pharyngeal pouch is 1–2% and usually smaller than primary pharyngeal pouch [1]. Histological evaluation of cricopharyngeal muscle in patients with ZD shows fibro-adipose tissue replacement and fibre degeneration which is keeping with impaired sphincter function of the cricopharyngeal muscle [3]. The most common type of pharyngeal pouch is the posterior pulsion diverticulum, but they can also be posterior-lateral or lateral only. Most pharyngeal pouches, approximately 90% are left sided [7] and histologically are lined with stratified squamous epithelium with fibrous submucosa. Rarely a squamous cell carcinoma or carcinoma in-situ may occur in the pouch, the prevalence is undetermined as there is limited data published [3,4,8].

Due to the proximity of the thyroid gland, ZD can occasionally mimic thyroid nodules and this can be misdiagnosed as a thyroid mass. Some diverticula contain fluid and debris which can project as complex thyroid cyst, haemorrhagic adenoma or even an abscess [9] and the ultrasound can show echogenic foci which can be seen as microcalcification [10] as evident in our case. To avoid this misunderstanding, repeat ultrasound is recommended or contrast radiography should be performed to exclude a pharyngeal pouch [9].

ZD's are usually diagnosed with a barium swallow or via an endoscopy [4,11]. Barium swallow is the most useful investigation to determine size and location of the diverticulum [2]. Treatment of pharyngeal pouch can be conservative or surgical treatment can be considered for symptomatic patients [1]. The surgical approach is either endoscopic (laser, stapling, and electrocoagulation) or external (inversion, diverticulectomy, cricopharyngeal myotomy). The gold standard is cricopharyngeal myotomy [6] however endoscopic surgery is becoming more prevalent. Endoscopic stapling of pharyngeal pouch was first described in 1993 and became a very attractive procedure for the treatment of pharyngeal pouch due to the brief operating time, low risk of morbidity and facilitation of early discharge from hospital [3,8,11] and is currently more favourable amongst UK surgeons as reported on the survey by Siddiq et al. [4,5]. A review performed in Oxford between 1992 and 2011 demonstrated the endoscopic approach had around 14% recurrence rate in a cohort of 320 patients [12]. The incidence of persistent or recurrent dysphagia is only 6% [4] and therefore the reported incidence of revision procedures is also low [3,8].

However, mortality rate is slightly higher after external approach (1–2%) which is partially due to increased age and frailty of these patients and partially due to the type of the surgery [1,11]. For histopathological examination of the pharyngeal pouch to exclude malignancy, diverticulectomy (external) is the only modality preferred [2]. Both treatment types are also used to treat recurrent pharyngeal pouch however reports show that endoscopic is preferred over the external approach. The endoscopic approach has shown to have improved symptoms associated with ZD without further complication, however this procedure is relatively new and complications may be underreported [3,4,7,11].

## 4 Conclusion

Recurrence of ZD following endoscopic surgery is a known complication and this should be considered as part of the differential diagnosis when investigating a large anterior neck mass. We recommend repeat imaging and considering an early CT scan when there is a suspicion or risk factors to exclude pharyngeal pouch.

## Provenance and peer review

Not commissioned, externally peer-reviewed.

## Ethical approval

Case report, not a research study.

## Source of funding

None.

## Author contribution

Dr. Fatemeh Salimi: Main author, Data collection, analysis.

Dr. Jerocin Vishani Loyala: 2nd Author.

Dr. Arbaz Perviaz: 3rd Author.

Professor Stuart Winter: Principle investigator, Design, data collection, analysis, interpretation, Contributing author.

## Declaration of competing interest

All authors have NO financial, personal nor conflicts of interest to disclose. All authors disclose that NO funding to be declared.

## Registration of research studies

1. Name of the registry:
2. Unique Identifying number or registration ID:
3. Hyperlink to your specific registration (must be publicly accessible and will be checked):

## Guarantor

Dr. Fatemeh Salimi.

Dr Vishani Loyala.
